# Prognostic value of metal-based ferroptosis and cuproptosis genes and score in lower grade gliomas

**DOI:** 10.3389/fimmu.2025.1608077

**Published:** 2025-09-02

**Authors:** Umair Ali Khan Saddozai, Zhendong Lu, Shuangshuang Dong, Muhammad Babar Khawar, Zhehao Fan, Liangliang Cai, Xiaohui Guo, Muhammad Usman Akbar, Saadullah Khattak, Haibo Sun, Yajun Wang

**Affiliations:** ^1^ Institute of Translational Medicine, Medical College, Yangzhou University, Yangzhou, Jiangsu, China; ^2^ Jiangsu Key Laboratory of Experimental and Translational Non-Coding Ribonucleic Acid (RNA) Research, Yangzhou, China; ^3^ Department of Medical Oncology, Beijing Tuberculosis and Thoracic Tumor Research Institute, Beijing Chest Hospital, Capital Medical University, Beijing, China; ^4^ Department of Pathology, Northern Jiangsu People’s Hospital Affiliated to Yangzhou, Yangzhou, Jiangsu, China; ^5^ School of Basic Medical Sciences, Faculty of Medicine, Yangzhou University, Yangzhou, Jiangsu, China; ^6^ Oujiang Laboratory; Key Laboratory of Alzheimer’s Disease of Zhejiang Province, Institute of Aging, Wenzhou Medical University, Wenzhou, Zhejiang, China; ^7^ Department of Preventive Medicine, Institute of Biomedical Informatics, Bioinformatics Center, Henan Provincial Engineering Center for Tumor Molecular Medicine, School of Basic Medical Sciences, Henan University, Kaifeng, Henan, China; ^8^ Department of Oncology, Haian Hospital of Traditional Chinese Medicine, Haian, Jiangsu, China

**Keywords:** ferroptosis, cuproptosis, cell death, immunotherapy, low-grade glioma, tumor microenvironment, immune checkpoint blockade

## Abstract

**Background:**

Ferroptosis and Cuproptosis are newly defined forms of cell death. Despite distinct mechanisms, both involve metabolic processes in the TCA cycle and downstream pathways, crucial for anticancer immunity.

**Methods:**

We evaluated Iron (Fe) and Copper-induced cell death in lower-grade gliomas (LGG) using The Cancer Genome Atlas (TCGA) data by developing a metal-based ferroptosis and cuproptosis genes score (MBFCGs) risk model. Lasso regression and survival analyses assessed MBFCGs’ significance. An MBFCGs-based nomogram was created and its predictive performance verified. Signaling pathways, immune checkpoints, chemokines, and therapeutic response indicators were quantified using R/oncoPredict and Tidepay. Immunohistochemistry (IHC) examined candidate gene expression.

**Results:**

The MBFCGs risk model, based on BACH1, CDCA3, and TIMP1, predicts LGG prognosis. High MBFCGs were associated with poor clinical outcomes. Functional enrichment analysis showed upregulation in neurotransmitter receptor regulation, KRAS signaling, and hedgehog signaling pathways in the high-risk group. High-risk LGG patients exhibited higher tumor mutation burden (TMB) and lower IDH1 mutation incidence. These patients also had increased stromal and immune scores, with elevated levels of T helper cells, B cells, macrophages, neutrophils, and NK cells. Immune checkpoint analysis indicated higher expression of CD274, PDCD1, and other inhibitory molecules, suggesting potential for targeted cancer immunotherapy.

**Conclusion:**

The MBFCGs risk model is a promising prognostic tool for LGG, offering insights into underlying mechanisms and new directions for immunotherapy strategies. Assessment of MBFCGs for individual LGG patients may provide clues for developing new immunotherapy strategies.

## Introduction

Gliomas are a class of tumors that mostly originate in the central nervous system and are well studied (1, 2). LGG is a subtype of Gliomas with a low malignancy, but they can still multiply and grow in a variety of ways. Consequently, the median overall survival for patients with grade II gliomas with LGG was only about 78 months (2). Therefore, it is essential to identifying the precise molecular trait for precise diagnosis, tailored care, and the prognosis of this disease.

Ferroptosis and Cuproptosis are two newly defined types of programmed cell death. Under normal physiological state, both Ferroptosis and Cuproptosis cell encounters are kept at extraordinarily low level by maintaining a dynamic balance to prevent accumulation of free intracellular Iron and Copper from harming cells (3). Ferroptosis linked to pathogenesis, development, therapeutic targets, and treatment resistance, is brought on by the accumulation of iron-dependent lipid peroxides at cell membranes (4, 5). This distinct non-apoptotic pathological cell death pathway has received more attention in recent years, particularly because of its potential tumor suppressor role (3, 6). Cuproptosis is a unique non-apoptotic programmed cell death initially proposed by the lab of Todd R. Golub in 2022 and found to be different from known cell death mechanisms like ferroptosis, pyroptosis, and necroptosis (7). The lipoylation proteins required to produce copper-induced cell death are primarily found in the tricarboxylic acid (TCA) cycle. Cell death, proteotoxic stress, and the loss of iron-sulfur cluster proteins are directly caused by copper binding to lipoylated proteins (7). Moreover, it may serve as a signal to facilitate reactions to the strengthened host defenses brought on immunological activation (8).

Recent research has demonstrated a clear correlation between the progression of cancer and high copper levels. Brady et al. showed that copper regulates the autophagic kinases ULK1/2, which is crucial in the development of lung adenocarcinoma (9). Mittal and colleagues also found that the triple negative breast cancer in mice is significantly suppressed when mitochondrial copper levels are low (10). The proliferation of cancer cells can be reduced by inhibiting the transport of copper (11).

Although ferroptosis and cuproptosis have been shown in numerous studies to be crucial in tumors, their influence on specific kinds of cancer is yet unknown. Interestingly, there are several steps involved in ferroptosis and cuproptosis, involving various pathways (canonical, noncanonical inflammasome, and alternative pathways). The overlap and potential crosstalk of the different pathways, characterizing the overall effects of metal-based ferroptosis and cuprotosis rather than cuproptosis and ferroptosis alone, could be a more effective strategy to understand their biological significance. Beyond their intrinsic roles in tumor cell regulation, ferroptosis and cuproptosis have garnered attention for their potential impact on the tumor immune microenvironment. In gliomas particularly LGG immune evasion is a key hallmark, and mounting evidence suggests that iron- and copper-dependent metabolic pathways may influence immune cell dynamics, tumor inflammation, and therapeutic sensitivity (5, 12–14). Notably, ferroptosis induction in tumor cells has been associated with enhanced CD8^+^ T cell-mediated cytotoxicity and improved responsiveness to immunotherapies (12, 15), while cuproptosis-related mechanisms have been linked to the regulation of cytokine secretion and redox homeostasis (7, 16).

Although ferroptosis and cuproptosis have been individually explored in various cancers, their integrated role in lower-grade glioma (LGG) remains poorly defined. To address this gap, we conducted a comprehensive analysis of genomic and transcriptomic alterations associated with these two metal-dependent cell death pathways. We hypothesized that a combined Metal-Based Ferroptosis and Cuproptosis Genes (MBFCG) score could effectively stratify LGG patients by prognosis and treatment response. Accordingly, we established and validated a prognostic risk signature based on the MBFCG score, highlighting its potential to predict survival outcomes, drug sensitivity, and the immunological landscape of LGG. This integrative approach offers novel insight into the clinical implications of ferroptosis and cuproptosis in LGG biology.

## Material and methods

### Dataset collection

Data on LGG gene expression and clinical details were sourced from Chinese Glioma ATLAS (CCGA) (http://cgga.org.cn/) and Cancer Genome Atlas (TCGA) (https://portal.gdc.cancer.gov/). In particular, TCGA-LGG has 529 samples, all of which have relevant clinicopathological features, copy number variations (CNV), single-nucleotide variants (SNV), and gene expression profiles. The values of the TCGA-LGG fragment were translated to transcript values per million. A uniform normalization and log2 transformation procedure was applied to all included RNA-seq data. A segmentation analysis utilizing the Genomic identification of Significant Targets in Cancer (GISTIC) algorithm was used to identify copy-number alterations as loss or gain levels.

Following the in-depth analyses, the SNV data were graphed using the R package “oncoplot” using the R package maftools. For external validation, two CGGA cohorts (CGGA1, 325 mRNAseq, RNA-seq, CGGA2, 301 mRNA array, Microarray) were obtained.

### Identification of metal-based genes and the construction of metal-based prognostic model

The FerrDB database (http://www.zhounan.org/ferrb/current/) (17) was used to retrieve ferroptosis-related genes while the cuproptosis-related genes were obtained from prior research by Jiang et al. (18). Using the R’limma and Pacman packages, we analyzed the correlation between cupproptosis and ferroptosis genes. Based on stringent screening criteria (P< 0.05 and correlation coefficient > 0.4), we identified 136 MBFCGs. Then, we performed Lasso-Cox dimension reduction analysis using the “glmnet” R package and applied univariate Cox analysis at P = <0.005, resulting in the identification of 24 critical genes. We then applied ten-fold cross-validation to carefully choose the optimal penalty parameter (λ) based on the minimum criterion to reduce the risk of overfitting. Subsequently, multivariate cox regression was conducted and identified three main genes from the pool of candidate genes. In the next step, we created a predictive model for MBFCGs using the three genes from the training cohort. The following formula was used to determine each sample’s MBFCGs: MBFCGs = ∑ (Coefi × Expi) , where “Exp” stands for each MBFCGs expression level and “Coef” for the coefficient.

### Evaluation and validation of the prognostic model

A prognostic scoring system was established for LGG patients, by utilizing the median value of predicted MBFCGs as the cutoff threshold.

We subsequently stratified patients into high-risk (MBFCGs > median value) and low-risk (MBFCGs< median value) groups, based on the distribution of MBFCG scores within the TCGA training cohort. Stratification thresholds were determined *a priori* to ensure consistency across validation datasets. (Page number 5 Line 110-118).

Kaplan-Meier analyses were conducted using the R packages “survival” and “survminer”. To measure the prognostic accuracy of the model, we conducted 1-,3- and 5-year receiver operating characteristic (ROC) analysis using the R package “timeROC,” and the area under curve (AUC) values were calculated. Calibration plots were generated using the bootstrap method with 1,000 resamples to assess agreement between predicted and observed outcomes, thereby validating the predictive performance of the MBFCG model.

To validate the metal-based prognostic model, we applied it to the internal testing cohorts and two external cohorts, CGGA1 and CCGA2. For each cohort of LGG patients, MBFCGs were calculated and the samples were divided into different risk groups. Subsequently, these groups underwent comprehensive assessments, Including Kaplan-Meier analysis, ROC analysis, and calibration analyses. Furthermore, we checked the survival time of all three datasets using the model MBFCGs by using Kaplan-Meier analysis.

### Tumor mutation burden, functional enrichment, and estimation of tumor microenvironment in cell infiltration

TMBs and Immune checkpoint genes (ICGSs) were associated with immunotherapy response rates. TMBs were associated with higher response rates, while ICGSs were associated with lower response rates. This suggests that TMBs and ICGSs may be important biomarkers for predicting response to immunotherapy. Early identification of TMBs and ICGSs can be used to identify patients who are more likely to respond to immunotherapy. This can help maximize the effectiveness of immunotherapy and improve patient outcomes. The “maftools” R program was used to extract the mutation annotation format (MAF) from the TCGA database in order to ascertain the mutational landscape of LGG patients based on the MBFCGs. In the whole TCGA cohort, the TMB score was also computed for every LGG patient. A GSEA was conducted to determine the gene sets that were statistically different between the high-risk and low-risk groups. Based on the MSigDB database, we used the gene sets “h.all.v7.2.symbols” and “c5.bp.v7.2.symbols.”. Enrichment of gene sets with an adjusted P-value of 0.05 was considered significant.

### Estimation of metal-based gene score in immunotherapy response

To estimate the immunotherapy response in LGG patients, the Tumor Immune Dysfunction and Exclusion (TIDE) algorithm (http://tide.dfci.harvard.edu) was employed. This algorithm assists clinicians in identifying patients who are better suited for immunotherapy (19).

### Prognostic independent analysis and nomogram establishment

We collected clinical characteristics such as age, tumor grade, and IDH mutation status of LGG patients within the entire TCGA cohort, and in two additional cohorts from Chinese Glioma Genome Atlas (CGGA). Combining these variables with the MBFCGs, we conducted both univariate and multivariable Cox regression analyses to examine their associations with patient survival outcomes. For personalized prediction of survival probabilities among LGG patients, we developed a nomogram using the clinical characteristics and the MBFCGs. This was accomplished using the “rms” and “regplot” R packages.

To evaluate the predictive accuracy of the model, we performed time-dependent Receiver Operating Characteristic (ROC) analysis for 1-,3- and 5-year survival probabilities.

In addition, calibration plots were generated to compare the predicted probabilities from the model with observed outcomes. These analyses were conducted in the TCGA-LGG cohort and validated in two additional cohorts, CGGA1 and CGGA2.

### MBFCGs correlation with immune infiltrates and immune checkpoint

To evaluate the composition of immune cell subsets within the tumor microenvironment, we employed the Cell-type Identification by Estimating Relative Subsets of RNA Transcripts (CIBERSORT) algorithm. This algorithm estimates the proportions of 22 immune cell types in bulk tumor samples based on gene expression matrices (20). To determine the relative number of immune cells, the TCGA-LGG RNA-Seq data was processed and normalized to transcripts per million (TPM). Subsequently, Spearman correlation analysis was conducted to explore the relationship between the abundance of infiltrating immune cells and MBFCG scores. P-values were adjusted using the FDR method to account for multiple comparisons. Then, we used Spearman’s rank correlation coefficient to examine the relationships between Immune Checkpoint Genes (ICGs) and the MBFCGs, and the three genes included in the MBFCGs prognostic model.

### Patients tissue samples for immunohistochemistry

A total of 26 cases of LGG were included in this study, encompassing individuals who underwent surgical resection at Northern Jiangsu People’s Hospital (Yangzhou, Jiangsu, China) between 2019 and 2023. Patients were selected based on inclusion criteria of histologically confirmed LGG, no prior chemotherapy or radiotherapy, and availability of sufficient tumor tissue. Sample size was determined based on availability during the specified time period, and no power analysis was conducted due to the exploratory nature of the IHC study. Ethical approval for this study was obtained from the Ethics Committee of medical college of Yangzhou University (Approval No. YXYLL-2024-101). Informed consent was obtained from all patients for the utilization of tissue samples for scientific investigation. Immediately following surgical excision, tissue specimens were promptly fixed in 10% paraformaldehyde.

### Immunohistochemistry

Paraffin-embedded sections, each 3 mm thick, were incubated at 95°C for 30 minutes in TE buffer (10 mmol/L Tris-HCl, pH 8.0, 1 mmol/L EDTA) to facilitate heat-induced epitope retrieval. Post-retrieval, the sections were incubated with primary antibodies anti-BACH1 (1:100 dilution, clone 9D11) or E-cadherin (1:200 dilution)—for 24 hours. Secondary antibody staining followed this incubation. The intensity of LGG staining was assessed based on BACH1 expression. IHC staining was independently evaluated by two blinded pathologists.

### Statistical analysis

All statistical analyses were conducted using R (version 4.3.2). For comparisons between groups (e.g., high-risk *vs*. low-risk), statistical significance was assessed using two-sided tests, including the log-rank test for survival curves and Wilcoxon rank-sum tests for continuous variables. A significance threshold of p< 0.05 was applied. Multiple hypothesis testing corrections were performed using the Benjamini-Hochberg method to control the false discovery rate (FDR), where applicable. Statistical significance was annotated as follows: *p< 0.05; **p< 0.01; ***p< 0.001; ****p< 0.0001.

## Results

### Identification of metal-genes and construction of metal-genes prognostic model

**Figure 1:**
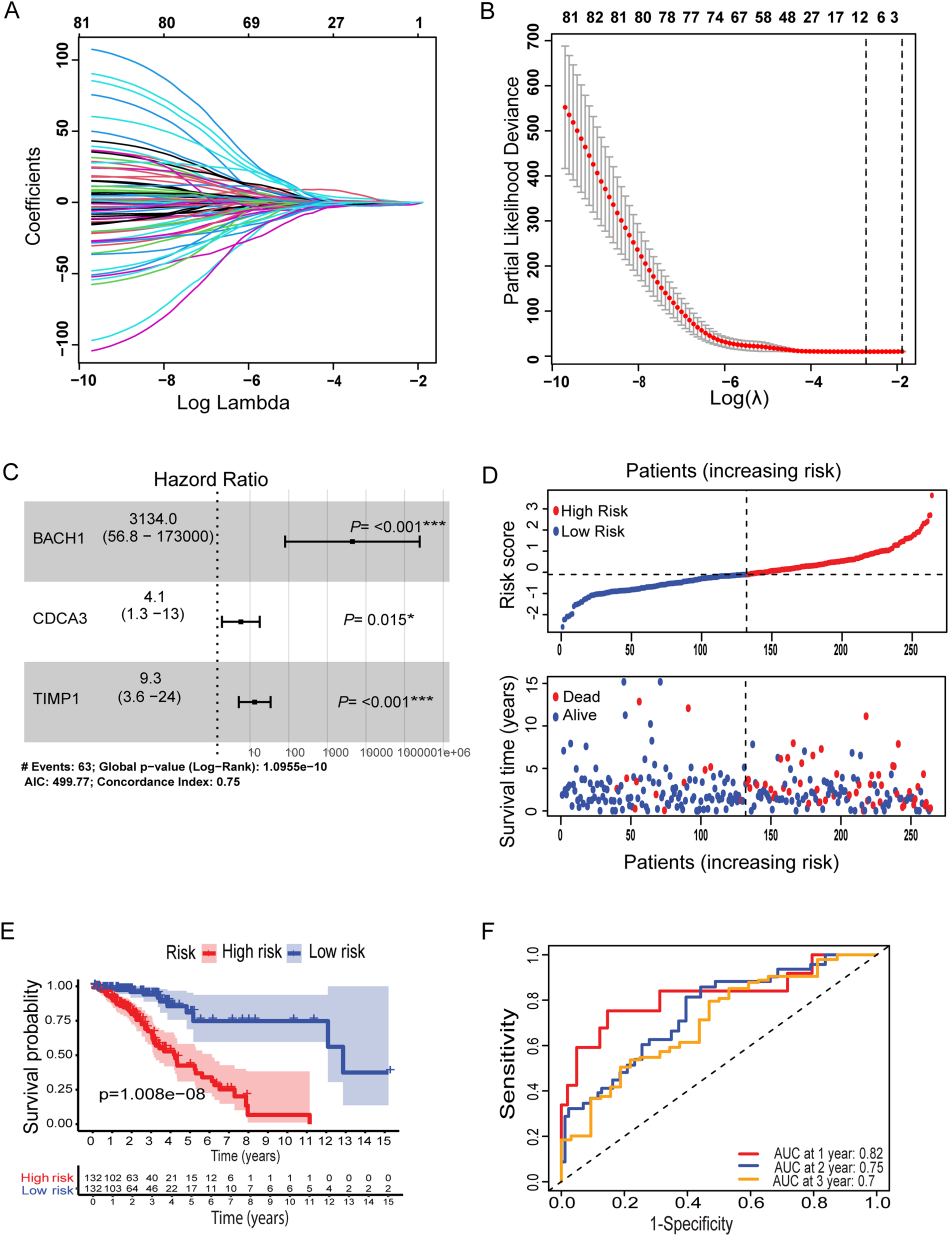
Lasso coefficient profiles of OS-related genes and cross-validation for tuning parameters selection in the LASSO regression **(A, B)**. Multivariate Cox regression analysis of three metal-based genes with overall survival in LGG patients **(C)**. Patient risk score distribution correlates with survival **(D)**. A Kaplan-Meier survival curve comparing high and low risk groups, showing survival probabilities over time **(E)**. **(F)** ROC curves for predicting sensitivity and specificity at one, two, and three years. Specifically, *: represents a p-value < 0.05, and ***: represents a p-value < 0.001).

To establish a fusion of the metal-genes dependent risk model in LGG, lasso and multivariate analyses were performed. Initially, the correlation analysis was performed between the metal genes of ferroptosis and cuproptosis. Further these genes were correlated with LGG-TCGA dataset which revealed a total of 134 metal genes. Patients in the TCGA cohort were divided into training and testing cohorts at a 1:1 ratio. Three main candidate genes were identified from the TCGA training cohort after lasso cox regression analysis and multivariate cox regression analysis. Based on these three genes BACH1, CDCA3, and TIMP1 metal-based prognostic model was constructed. The associated metal-genes score can be calculated as follows; Metal-gene score= 1.6265423* BACH1 + 6.0534006 CDCA3 + 0.4718346 TIMP1.The Forest plot, Hazard ratio (HR), and significance are shown in the overall prognosis (**Figures 1A–C**). The risk plot showed that patients with a higher metal-gene score had more deaths and shorter survival times (**Figure 1D**). The Kaplan-Meier analysis showed that high-risk patients had worse overall survival than low-risk patients in both TCGA testing and training groups (P = 1.008e-08) (**Figure 1E**).

**Figure 2:**
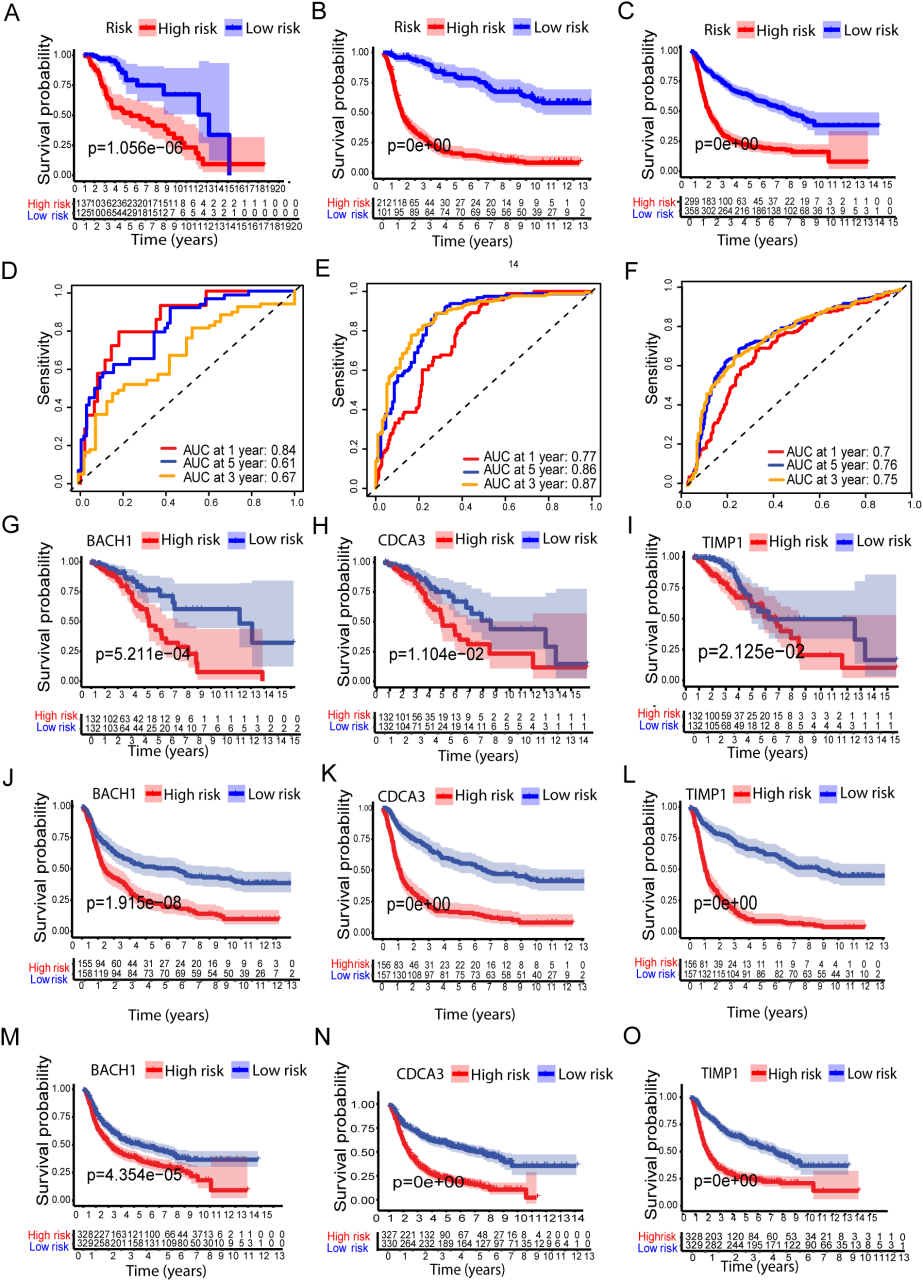
Validation of the Metal-based prognostic model in the TCGA, CCGA1, and CCGA2 datasets. **(A–C)** represents Kaplan–Meier overall survival (OS) curves for high- and low-risk groups for TCGA, CCGA1, and CCGA2 datasets, respectively. While **(D–F)** ROC curves, depicting prognostic performance. **(G-O)** show survival analysis of the metal-related genes BACH1, CDCA3, and TIMP1 in high- and low-risk groups across the TCGA, CCGA1, and CCGA2 datasets.

The survival probability of the metal-genes score in TCGA training was represented by the AUC values of 0.82, 0.75, 0.7 respectively for 1-,3, and 5-years, indicating good parameters of the accuracy of modeling (**Figure 1F**). Similarly, Kaplan-Meier analysis revealed a significant difference in overall survival between high- and low-risk patients (**Figure 2A**), while the AUC values for 1-, 3-, and 5-year predictions in the TCGA testing cohort were 0.84, 0.61, and 0.67, respectively (**Figure 2D**). In addition, the calibration curve showed a satisfactory agreement between the predictive and observational values at the three and five-year time periods (**Supplementary Figures S1A–D**). Taken together, the risk plot the metal gene score and Kaplan-Meier survival revealed that the patient with the high-risk score has more cases of death and shorter survival time in both TCGA training and testing cohorts (**Figure 2D**).

### Metal-genes based prognostic model external validation

To further validate the prognostic performance of the model, we conducted validation using two external cohorts, CCGA1 and CCGA2. Subsequently, patients were stratified into high and low risk groups based on metal-genes scores. Kaplan-Meier analysis demonstrated improved prognosis within the high-risk group compared to the low-risk group (**Figures 2B, C**).

Furthermore, the model exhibited a notably high AUC value in the external validation cohorts, as shown in (**Figures 2E, F**). Additionally, Kaplan-Meir analysis of all three metal genes shows significant differences in both risk groups of all three datasets of LGG. The high-risk group showed significantly lower survival of Kaplan-Meier analysis (KMP) in all three datasets of LGG when tested separately with all three genes (**Figures 2G–O**).

### Functional enrichment, tumor mutation burden and estimation of tumor microenvironment cell infiltration

**Figure 3:**
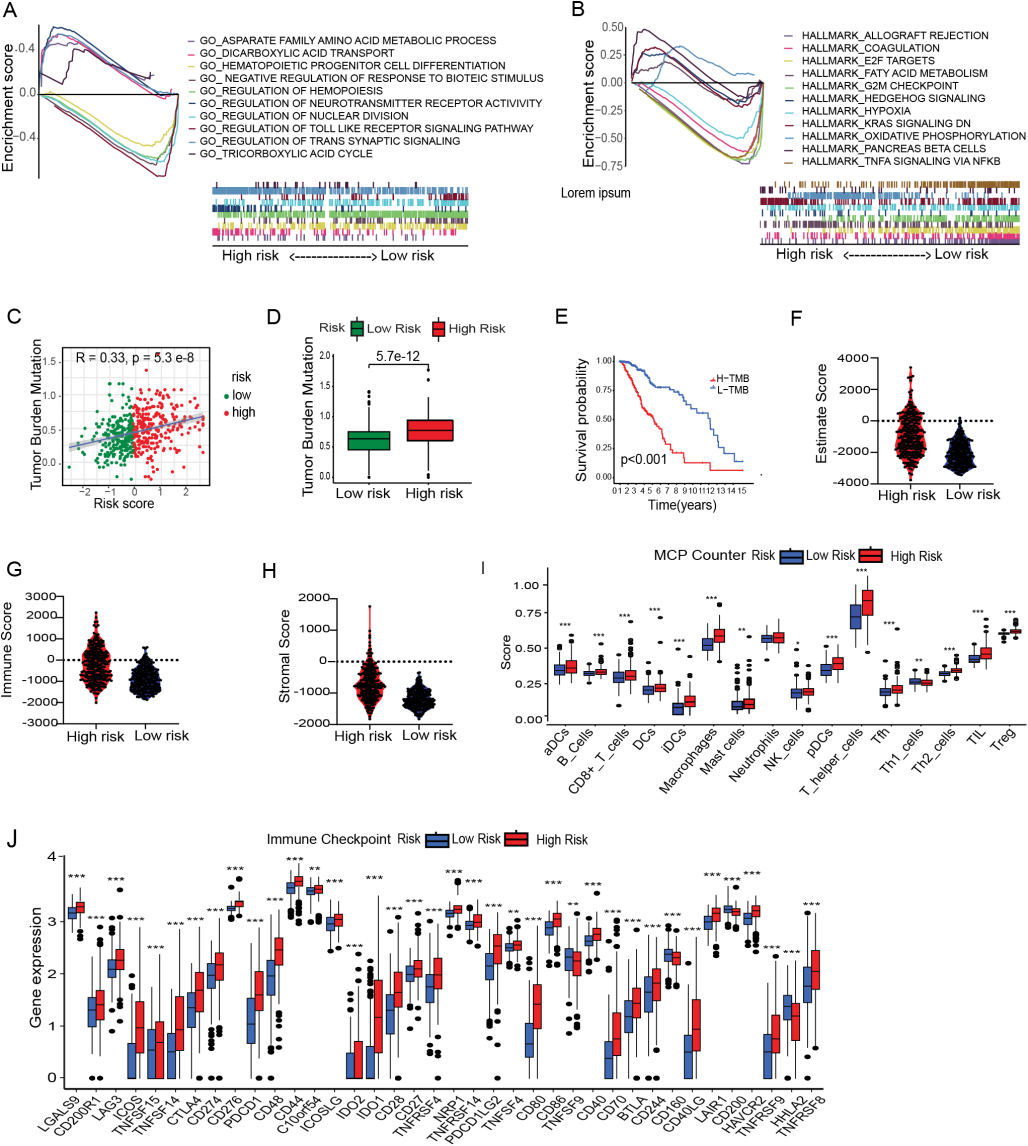
Immune correlation with Metal-genes scores and cancer stem cell index. GSEA reveals the significant GOBP and HALLMARK terms enriched in the High and Low risk Group **(A, B)**. Correlation between MBFCG score and TMB. High and low risk difference based on TMB **(C, D)** and survival of the patients based on high and low TMB **(E)**. Comparison of Immune Score, Stromal score and ESTIMATE score between the two risk group **(F–H)**. Analysis of differences in immune cell abundance between risk groups using MCP counter **(I)**. Expression level of immune checkpoints of risk groups, *p<0.05, **p<0.01, ***p<0.001 **(J)**.

To gain insight into the underlying biology contributing to the remarkable predicative ability of the metal-gene score, we performed GSEA enrichment analysis in the TCGA-LGG high and low-risk group. GO analysis showed significant upregulation of pathways related to the regulation of neurotransmitter receptors, regulation of trans-synaptic signaling, and dicarboxylic acid transport in the high-risk metal-genes score group. While the HALLMARK showed the overexpression of KRAS signaling, pancreas beta cells hallmark, and hedgehog signaling (**Figures 3A, B**).

The tumor is surrounded by a variety of immune cell types that infiltrate the dynamic microenvironment. These immune elements interact with tumor cells to influence the immunogenicity of tumors and their susceptibility to checkpoint inhibitors, including anti-Programmed cell death (PD-1) and anti-PD-L1 antibodies. We next looked into the relationship between the metal-genes score and immunological infiltration, taking into account the different immune high and low metal-gene score groups. Prior research has demonstrated a significant correlation between elevated TMB scores and enhanced immunotherapy response. A significant correlation was found between the metal-gene score and TMB (R = 0.33, P = 2.3e-8), and the TMB scores were increased significantly (P = 5.7e-12) in the high-risk group in the current investigation. The KMP illustrates the survivability between the two groups (**Figures 3C–E**). Moreover, we found that high-risk group, (66%) had a substantially lower incidence of Isocitrate dehydrogenase 1 (IDH1) mutations compared to those in the low-risk category of (90%). Similarly, TP53 and IDH1 mutations were crucially correlated with the increased Metal-gene Score in (**Supplementary Figures S1E, F**). These results suggested that metal-gene score and IDH1 mutations may potential Metal-dependent apoptotic activity in LGG.

Tumor immunity plays a key role in tumor growth and survival of the patients. Hence, we examined the tumor immunity risk groups of LGG. The ESTIMATE package was used to infer the stromal score and immune score of LGG specimens. High metal-gene scores were associated with increased stromal and immune scores (**Figures 3F–H**), indicating enhanced stromal and immune cell levels in LGG TME. Multiple immune infiltrates were elevated in tumors with high metal-gene scores. These results suggest the potential differences in tumor immune response between high-risk and low-risk groups. Therefore, we used the MCP counter and TIMER to analyze related immune factors, such as immune infiltration and immune checkpoints. Specifically, compared to the low-risk groups, the high-risk group showed significantly higher levels of T helper cells, B cells, macrophages, neutrophils, and NK cells (**Figure 3**). Multiple immune checkpoint expression including CD274, PDCD1, BTLA, CTLA4, CD276, HAVCR2, and LAG3 uncovered the significant overexpression in the high-risk group (**Figure 3J**). These inhibitory checkpoint molecules protect tumors from damage and attack, making them promising targets for cancer immunotherapy. A prominent accumulation of T helper cells and macrophages in the high-risk group indicates the presence of an immunosuppressive TME, likely shaped by persistent inflammation and the recruitment of tumor-associated macrophages (TAMs). Concurrently, increased infiltration of neutrophils and NK cells may reflect the activation of innate immune responses; however, their precise functional roles within the LGG microenvironment remain poorly defined. Notably, although NK cells are more abundant in high-risk cases, their cytotoxic activity may be attenuated by functional exhaustion, potentially mediated by the upregulation of inhibitory immune checkpoint molecules such as CD274 (PD-L1) and PDCD1 (PD-1). These findings suggest that high-risk LGGs may adopt immune evasion strategies through checkpoint pathway modulation and TME remodeling, thereby dampening antitumor immune responses and contributing to unfavorable clinical outcomes.

### Estimation of metal-based gene score in immunotherapy response

**Figure 4:**
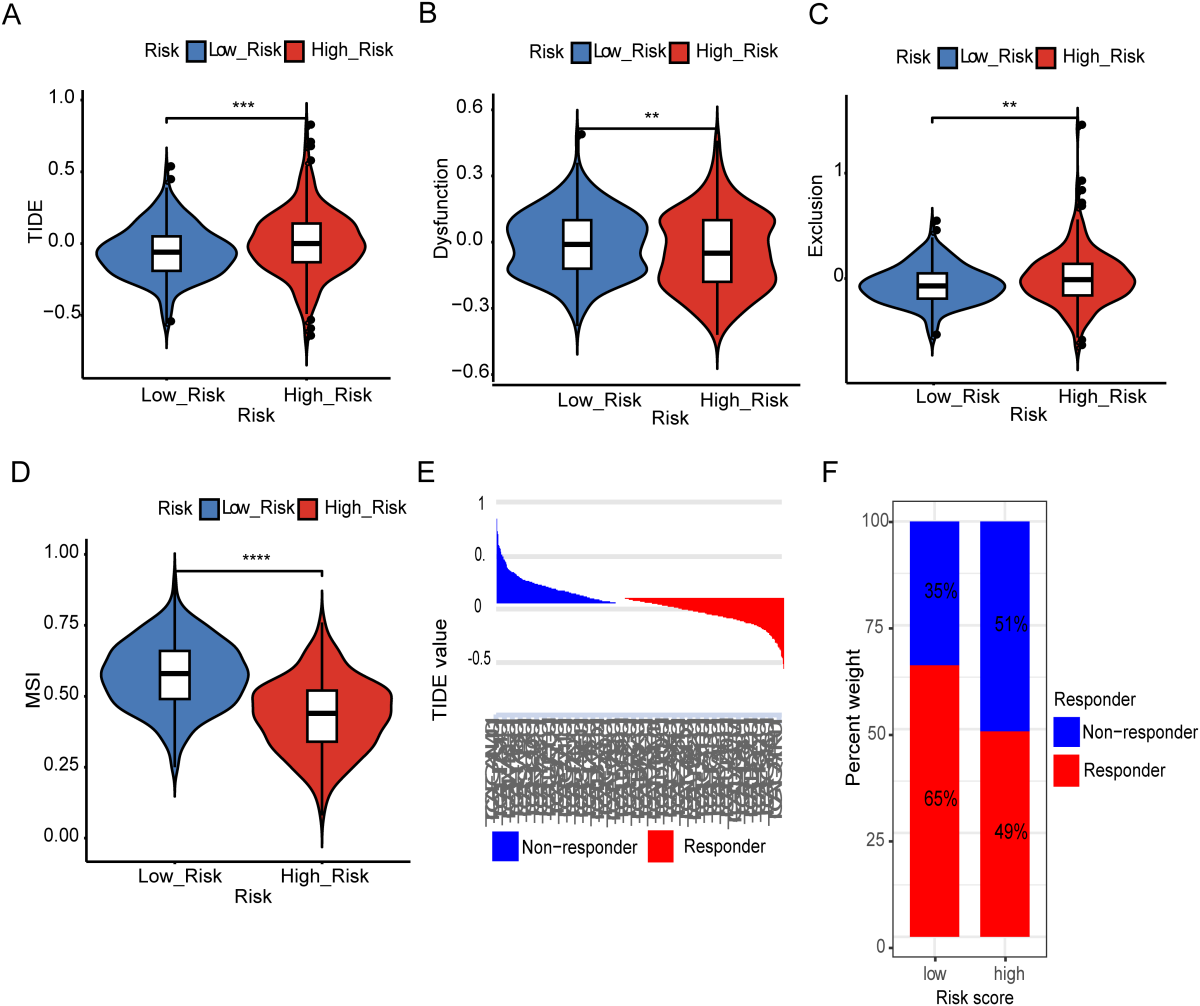
Estimation of Metal-based prognostic model in immunotherapy response. TIDE scores difference in between both risk groups **(A)**. Difference in dysfunction score between high and low risk groups **(B)**. Difference in exclusion between two risk groups **(C)**. Representing difference in MSI between two risk groups **(D)**. TIDE value and the proportion of clinical response in the low and high-risk group of TCGA cohort **(E, F)**. Specifically, **: represents a p-value < 0.01, and ***: represents a p-value < 0.001. ****: represents a p-value < 0.0001).

To our best knowledge, no research work has been previously reported on metal-immunity interactions, including Fe and Cu deficiency. The role of metal-dependent apoptosis in immune-oncology has hardly been investigated. To better understand the relationship between metal-gene scores and immunotherapeutic responses, we evaluated predicted immunotherapy responsiveness using transcriptome data from patients with LGG using the Tumor Immune Dysfunction and Exclusion (TIDE) method. According to the results, the high-risk group had a much higher TIDE score than the low-risk group, which may indicate the lower-risk group is benefited more from immunotherapy. The TIDE, dysfunction and exclusion scores showed significant differences: individuals in the low-risk group scored lower on exclusion and higher on dysfunction (**Figures 4A–C**). However, the low-risk group had a higher Microsatellite instability (MSI), representing a better prognosis (**Figure 4D**). Subsequently, the TIDE algorithm classified the patients as responder and non-responders, revealing once more an intriguing correlation between immunotherapy responsiveness and a lower metal-gene score (**Figures 4E, F**). These findings provide insights into the relationship between metal-gene interactions and the responsiveness of LGG patients to immunotherapy.

### Estimation of metal-based gene score in chemotherapy response

**Figure 5:**
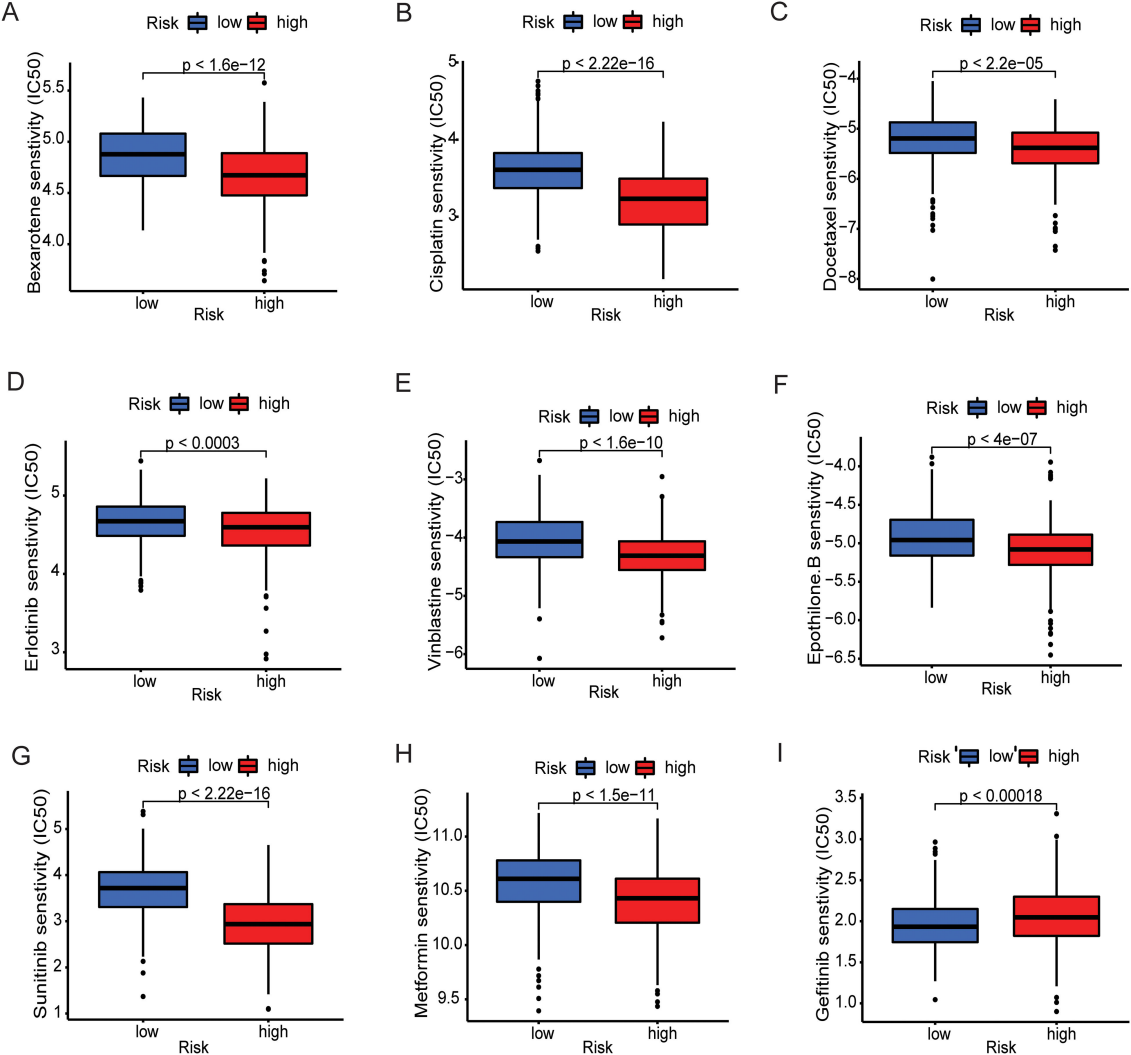
Prediction of therapeutic sensitivity in MBFCG high and low risk for the TCGA patients. Differences in estimated IC50 values of common drugs for high and low risk groups. **(A–H)** show the elevated level of IC50 in low-risk group indicating lower predicted drug sensitivity. **(I)** Representing the Gefitinib only shows the lower sensitivity to low-risk group.

To determine the value of the metal-gene score as a biomarker for anticipating chemotherapeutic responsiveness in LGG patients, we assessed the association between our risk model and the sensitivity of prevalent chemotherapeutic agents. This analysis involved analyzing the Half-maximal inhibitory concentration (IC50) values through the utilization of the “oncoPredict” R package, differentiating between the high-risk and low-risk cohorts. Different agents used in the clinic for cancer and specifically for LGG including cisplatin, imatinib and vinblastine showed consistently higher sensitivity in the lower group. Only gefitinib had higher IC50 values in the high-risk group which makes a clear distinction between the groups and provides one new door of specific drug for the patients of high-risk (**Figure 5**).

**Figure 6:**
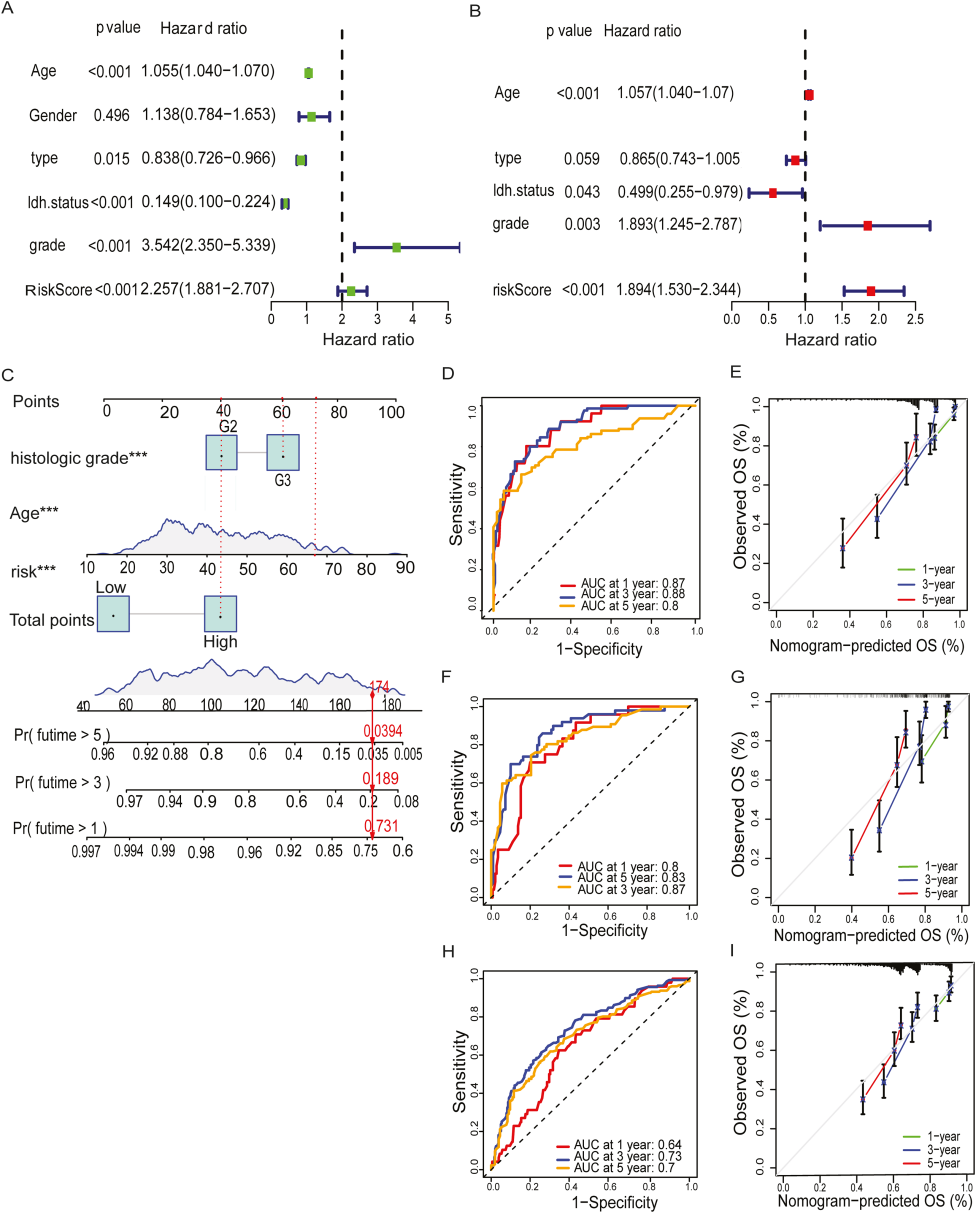
Establishment of nomogram and independent prognostic analysis. **(A, B)** Univariate and Multivariate Cox regression analysis of LGG and the clinical characteristic in the TCGA cohorts. **(C)** A nomogram was developed to predict 1-,3- and 5-year overall survival ability of LGG training cohort. **(D)** ROC curves showed the prognostic performance of the model in the TCGA cohort. **(E)** The calibration curves quantified the correlation between the model’s anticipated and actual results for the TCGA cohort. **(F)** ROC curves demonstrated the model’s prognostic performance in the CGGA1 cohort. **(G)** The calibration curves assessed the correlation between the model’s anticipated results and the observed results for the cohort known as CGGA1. **(H)** ROC curves demonstrated the model’s prognostic performance in the CGGA2 cohort. **(I)** The calibration curves assessed the correspondence between the model’s anticipated and observed results for the CGGA2 cohort. Specifically, ***: represents a p-value < 0.001).

To assess the predictive performance of the MBFCGs model, we constructed a nomogram integrating clinical parameters with the risk score and evaluated its accuracy across multiple cohorts. Univariate and multivariate Cox regression analyses were conducted to assess the independent prognostic value of the MBFCG risk score alongside clinical parameters. In TCGA dataset univariate analysis identified age, type, IDH status, histologic grade, and the MBFCG risk score as significant predictors of OS all p< 0.001 (**Figure 6A**). Similarly, in multivariate analysis, the MBFCG risk score remained an independent prognostic factor (p < 0.001), even after adjusting for age, tumor type, and histological grade (**Figure 6B**). Finally, the overall survival probabilities at 1, 3, and 5 years were predicted by the nomogram using the biomarkers Histological grade, Age, and score expression levels, contributing to the total score (**Figure 6C**).

To assess the predictive accuracy for prognosis, AUC values were used to assess the model’s discriminative performance. In the TCGA dataset the accuracy of AUC for 1-, 3-, and 5-year were 0.87, 0.88, and 0.8, respectively (**Figure 6D**). It is noteworthy that the model demonstrated robust predictive capability in the CGGA1 cohort (AUC > 0.75) (**Figure 6F**), and in the CGGA2 cohort (AUC > 0.7) (**Figure 6H**). Calibration plots further demonstrated a high degree of agreement between the model predicted capabilities and observed outcomes, as depicted in (**Figures 6E, G, I**).

### MBFCGs correlation with immune infiltrates and immune checkpoint

**Figure 7:**
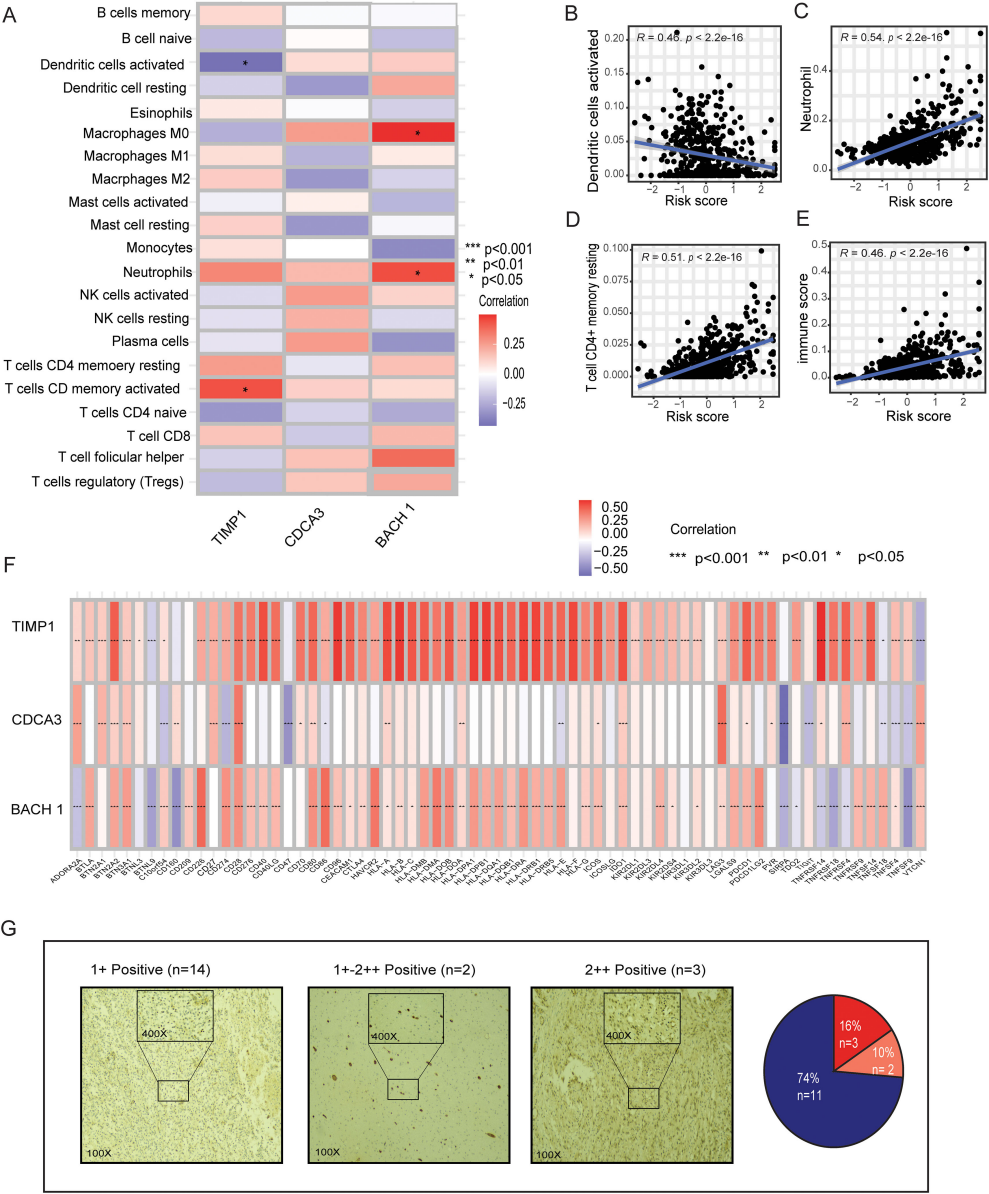
Relationships between immune infiltration and ICGs in LGG and the MBFCG score. **(A)** Relationships in the metal-based prognostic model between three genes and immune cell abundance. **(B–E)** Correlations between the MBFCG score and immune cell abundance. **(F)** ICG expression correlations with three genes in the metal-based prognostic model. **(G)** IHC analysis of BACH1expression in human LGG samples.

Given the distinct immune characteristics observed in high and low metal-gene score, we delved into the association between metal-gene score and immune infiltration. We further explored the correlation of immune infiltration and immune and immune checkpoints with 3 genes of metal-genes related prognostic models (**Figure 7A**). The metal-gene score showed a negative correlation with activated dendritic cells, but a positive correlation with macrophages M0, neutrophils, and T cells with CD memory activation (**Figures 7B–E**). We also investigated the connection between metal-gene score and ICGs, since it has been demonstrated that ICG expression levels are correlated with the therapeutic benefit of checkpoint blockade immunotherapy (**Supplementary Table 4**). A noteworthy association was discovered between the majority of the ICGs and the three genes. The metal-gene score was shown to be negatively correlated with VTL9, SIRPA, and CD47; nevertheless, the high-risk patient showed an increase in the expression level of 69 ICGs, including CD276, BTN2A2, PDCDILG2, CD274, and CD276 (**Figure 7F**).

### Immunohistochemistry of BACH1

IHC analysis of clinical specimens was conducted to explore BACH1 expression. To achieve this, we developed a monoclonal antibody (mAb) targeting human BACH1. Interestingly, all the samples found to have the Positive expression of BACH1. Particularly, patients were categorized into three groups based on the intensity of staining such as +weak, +-++moderate, and ++strong. Out of 19 samples 14 found to be + (74%) weak, 2 samples had a +-++ moderate (10%) and 3 samples showed strong staining intensity of ++(16%). Overall, the IHC result indicates it as a new biomarker for LGG patients for the further confirmation (**Figure 7G**).

## Discussion

The term “ferroptosis” was coined in 2012, and there has been observed an exponential growth in ferroptosis research in recent years. Increasing evidence indicates that ferroptosis may play a significant physiological role in both immunity and tumor suppression (12, 21). Additionally, a growing body of research indicates that copper plays a critical role in processes such cancer spread, tumor angiogenesis, and cell proliferation (22, 23). Numerous studies have delved into cuproptosis and ferroptosis in the context of cancer. However, the collective impact on cancer, particularly on LGG, remains incompletely characterized. Consequently, the role of the metal-genes score in the development of the TME and its potential therapeutic significance remains unclear. Hence, it is reasonable to assume that a comprehensive assessment of metal-dependent apoptosis gene status could optimize therapeutic outcomes and facilitate the development of synergistic treatment approaches.

To enhance the evaluation of metal-gene patterns in individual LGG patients, a comprehensive analysis of metal-related genes was conducted (**Supplementary Tables 1, 2**). This led to the identification of three key metal genes BACH1, CDCA3, and TIMP1 from which a predictive metal-gene-related prognostic model was constructed. Using lasso-cox risk scores, patients were stratified into high and low-risk groups. Notably, the low-risk group exhibited significantly longer overall survival time, consistently validated across both CCGA cohorts. This suggests that a high-risk score may serve as an indicator of an unfavorable prognosis. The roles of our identified metal-related genes, BACH1, CDCA3, and TIMP1, were thoroughly examined in the context of previous studies.

BACH1, a CNC-bZip transcription factor (24), regulates iron-related genes and promotes breast cancer metastasis (25). In glioblastoma, elevated BACH1 correlates with an immunosuppressive microenvironment, suggesting therapeutic potential (26). However, BACH1’s role in LGG remains unclear, necessitating further exploration. Often overexpressed in tumor tissues, CDCA3 is linked to carcinogenic qualities in a number of malignancies, such as gastric (27), non-small-cell lung (28), prostate (29), and colorectal (30). Numerous studies consistently show significant TIMP1 upregulation across various cancers (31, 32). Meta-analyses reveal plasma TIMP1 as an independent prognostic marker in certain cancers (33, 34). Notably, in LGG, elevated TIMP1 expression correlates with a poorer prognosis (35).

In our study, GSEA enrichment analysis unveiled the pivotal involvement of biological processes in distinguishing between patients with high and low-risk metal-gene scores. The GO analysis elucidated the high expression of the tricarboxylic acid cycle (TCA) within the high-risk group. The significance of the TCA cycle was found to be intricately linked with the cuproptosis type of cell death (7). Furthermore, HALLMARK enrichment analysis demonstrated that the high-risk group predominantly exhibited enrichment in hedgehog signaling and KRAS signaling. As a result, we postulate that the changes in survival outcomes between the two groups may be attributed to the activation of hedgehog and KRAS signaling pathways (**Supplementary Table 3**). Despite a decade of progress in LGG therapy, significant gaps persist, with treatment response heterogeneity. Standard multimodal approaches (36) face challenges in addressing diverse patient outcomes (37). Mansouri et al.’s study highlights MGMT promoter methylation’s predictive accuracy for temozolomide chemotherapy outcomes (38). Further exploration of intratumor heterogeneity and TMB’s impact on prognosis is crucial. The potential benefit of IDH1/2 mutations in upregulating VEGF and HIF-1α is noteworthy (39). Consistent with previous studies, our data unveils a notable contrast in TMB between metal-gene score subgroups. Patients, in the high-risk group, show the mutation of TP53, EGFR, and PTEN are observed (**Supplementary Figure 1**), ultimately known to correlate with poorer clinical outcomes (40). We continued our study to evaluate the efficacy of immunotherapy in various LGG subgroups. Our research showed that there were differences in 38 immunological checkpoint expression between the two groups. Interestingly, most immunological checkpoints including (LAG3, CTLA4, CD274, and PDCD1) were over-expressed in the high-risk group. In contrast, the low-risk group only showed increased expression of IDO1, TNSF9, and CD160. This suggests a prevalence of multiple inhibitory mechanisms within the high-risk group, thereby emphasizing the potential efficacy of targeted immunotherapeutic interventions specifically tailored for high-risk group of LGG. Understanding these distinct immunological profiles could significantly inform prognosis and treatment strategies, potentially leading to improved survival outcomes for patients with LGG.

Furthermore, the TME features showed that a higher metal-gene score was correlated with a higher stromal score and a higher ESTIMATE score. An increasing body of research highlights the critical function of various immune cells in the immune response against LGG (41). The three metal-gene profiles also showed a strong correlation with immune cells. Additionally, a higher metal-gene score showed a negative correlation with active dendritic cells alone, but a positive association with neutrophils, activated T cells, and macrophages M0. Targeting these immune checkpoints may assist patients even more for successful immunotherapy techniques, given the possible association between the aforementioned hallmark genes and immune checkpoints. Furthermore, drug sensitivity analysis enabled the metal-genes risk model to predict candidate drugs. The majority of drug candidates, such as cisplatin, gefitinib, and vinblastine, show promise in the low-risk group, suggesting a higher likelihood of treatment response and improved clinical outcomes.

Immune infiltration profiling revealed a substantial enrichment of T helper cells, macrophages, neutrophils, and NK cells in the high-risk group, indicating an inflamed yet immunologically dysfunctional TME. While such immune-rich TMEs often suggest heightened immunogenicity, the concurrent upregulation of inhibitory immune checkpoints including PDCD1, CD274, CTLA4, and LAG3 points to a state of immune exhaustion. Although immune cells are actively recruited, their effector functions appear attenuated by checkpoint-mediated suppression. Additionally, the elevated presence of M0 macrophages and neutrophils likely contributes to the establishment of an immunosuppressive niche. Particularly, TIMP1 has been implicated in promoting pre-metastatic niche formation via neutrophil recruitment through the SDF1/CXCR4 axis (42). CDCA3 expression correlates with immune exhaustion markers such as PD-1 and CTLA-4, and is associated with increased immune infiltration and poor outcomes in renal and lung cancers (43, 44). Moreover, BACH1, particularly through the BACH1-IT2/miR-4786/Siglec-15 axis, has been shown to mediate immune evasion by enhancing Siglec-15 expression and suppressing immune activation (45). Finally, we created a quantitative nomogram for prognostic classification in LGG patients to improve performance and streamline the implementation of the metal-gene score.

Nomograms have emerged as powerful statistical tools for predicting patient outcomes across various cancers. By integrating multiple clinical or molecular variables, nomograms enable individualized risk stratification and often surpass traditional stage-based systems in predictive accuracy. Their application mitigates subjective bias and offers clinical guidance, particularly in cases where the potential benefit of additional treatment remains uncertain (46–49).

In recent years, the prognostic value of nomograms has garnered increasing attention in LGG research. Gittleman et al. developed a nomogram using TCGA data, including age, WHO grade, Karnofsky performance status, and IDH-based molecular subtype; this model was externally validated in the Ohio Brain Tumor Study cohort, offering reliable individualized survival estimates (50). Similarly, Han et al. adapted and validated a refined version in a large Chinese Glioma Genome Atlas (CGGA) cohort (n = 582), incorporating age, tumor grade, molecular subtype, and post-operative treatment, confirming its applicability in Asian populations (51). More recently, Guo et al. introduced a DNA methylation-driven nomogram combining epigenetic markers (ARL9, CMYA5, STEAP3) with clinico-molecular features (IDH1 mutation, age, sex), which demonstrated outstanding prognostic performance (AUC = 0.93) in both TCGA and external CGGA validation cohorts (52). These models underscore the increasing sophistication and predictive accuracy of nomogram-based tools in LGG. Extending these findings, we conducted a systematic analysis of MBFCGs in LGG biology and established a novel significant risk model based on three MBFCGs genes (BACH1, CDCA3 and TIMP1). These uncovered distinct pathways, immune infiltrative characteristics and the better prognostic model.

This model demonstrated strong predictive accuracy, with AUC for 1-, 3-, and 5-year were 0.87, 0.88, and 0.8, for 1-, 3-, and 5-year survival, respectively in the TCGA dataset. It is noteworthy that the model demonstrated robust predictive capability in the CGGA1 cohort (AUC > 0.75), and in the CGGA2 cohort (AUC > 0.7).

Notably, the inclusion of BACH1 as a novel biomarker add depth to our understanding of LGG pathogenesis and offer a potential avenue for targeted therapeutics interventions.

MBFCGs related genes are gaining traction as clinically relevant markers in glioma stratification. The recent identification of cuproptosis as a distinct form of programmed cell death characterized by copper-induced lipoylated protein aggregation and proteotoxic stress offers novel mechanistic insights that extend beyond traditional apoptosis or necroptosis (7). Similarly, emerging clinical data suggest that dysregulation of copper homeostasis and cuproptosis-related pathways can influence treatment sensitivity and tumor immune evasion, highlighting cuproptosis as a potential therapeutic target in cancers such as esophageal carcinoma (18). Although the clinical application of cuproptosis-related biomarkers remains in its early stages, emerging evidence suggests that key regulators such as FDX1 hold promise as prognostic indicators and therapeutic targets in cancer. These biomarkers have the potential to guide patient stratification and inform personalized treatment strategies by predicting tumor sensitivity to copper-modulating therapies. Nonetheless, further clinical validation and standardized testing protocols are necessary before widespread implementation in routine practice can be realized (7).

In our study, the MBFCG model, by integrating both ferroptosis and cuproptosis genes, may serve as a biomarker tool to stratify LGG patients into immunotherapy-responsive and chemotherapy-sensitive subtypes. Given the differential TMB levels, immune checkpoint profiles, and drug response signatures observed between MBFCG-defined risk groups, the model could be applied in routine clinical workflows for risk stratification and therapeutic planning. Further validation in prospective, multi-center cohorts and clinical trials evaluating MBFCG targeting agents will be essential to fully translate these biomarkers into practice.

In conclusion, our findings provide a promising framework for guiding personalized therapeutics strategies in LGG patients, paving the way for more personalized and effective treatment.

This study also has few limitations. The MBFCGs risk model was constructed using retrospective data derived from publicly available cohorts, which may inherently introduce selection bias. Consequently, its validation in prospective, multi-center cohorts and clinical trials evaluating MBFCG targeting agents will be essential to fully translate these biomarkers into practice. Furthermore, our findings highlight the potential functional role for MBFCGs genes, mechanistic confirmation through *in vivo* and *in vitro* assay remains essential to full elucidate their biological relevance in LGG.

## Data Availability

The original contributions presented in the study are included in the article/**Supplementary Material**. Further inquiries can be directed to the corresponding authors.
