# Supplementary material for: Prognostic value of metal-based ferroptosis and cuproptosis genes and score in lower grade gliomas
Source: Front Immunol. 2025 Sep 2;16:1608077. doi: 10.3389/fimmu.2025.1608077 (PMC12436455; doi:10.3389/fimmu.2025.1608077)
Supplement: Supplementary file 1 [file DataSheet1.pdf]

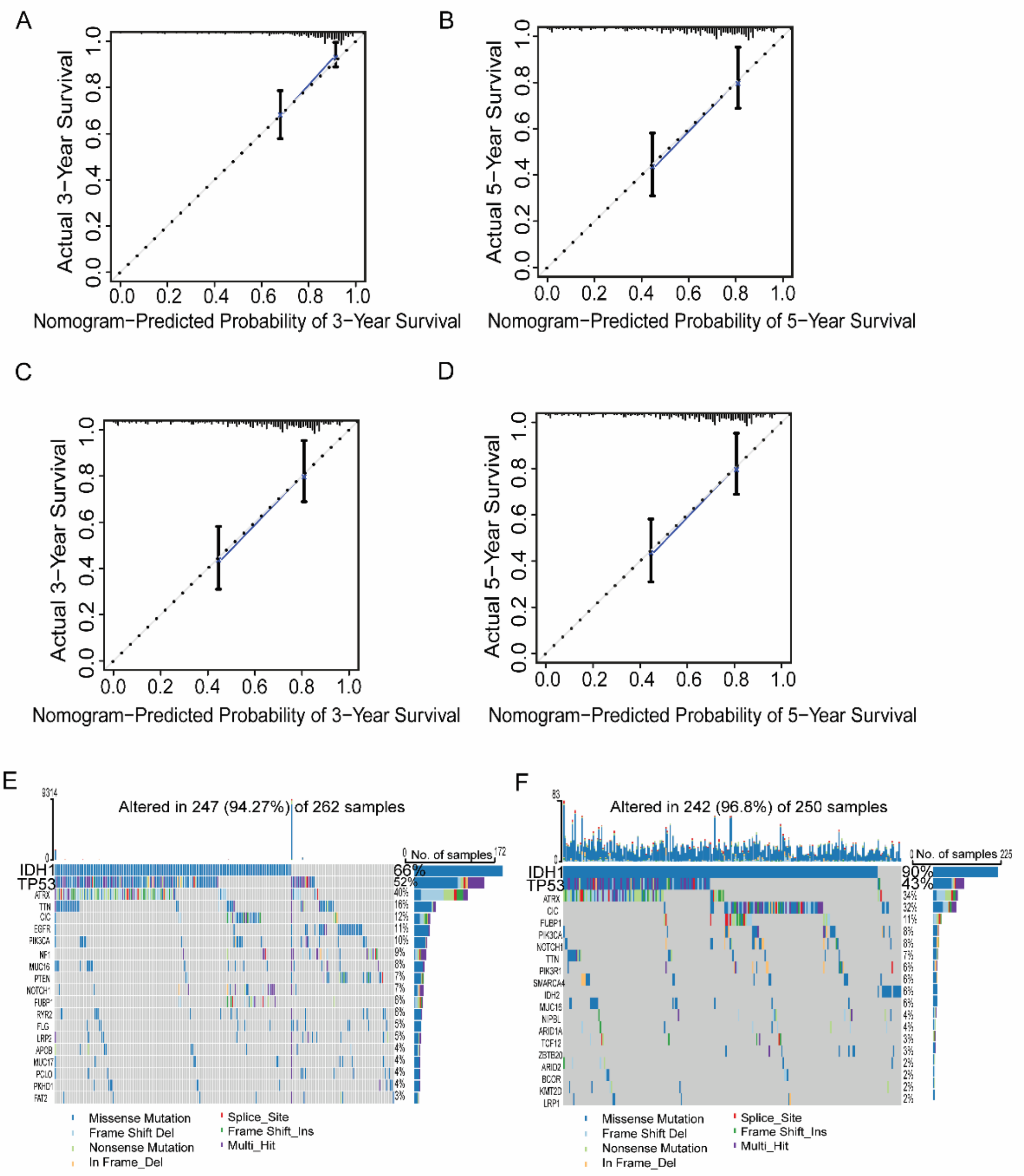

Supplementary Figure 1. The calibration curve showed a satisfactory agreement between the predictive (A, B) and observational values (C, D) at the three and five-year time periods. While (E, F) representing the differences TMB mutation of TP53, EGFR, and PTEN between metal-gene in high-risk and low risk groups.
